# Years of life lost during the Covid-19 pandemic in Sweden considering variation in life expectancy by level of geriatric care

**DOI:** 10.1007/s10654-022-00915-z

**Published:** 2022-09-20

**Authors:** Marcus Ebeling, Enrique Acosta, Hal Caswell, Anna C. Meyer, Karin Modig

**Affiliations:** 1 Unit of Epidemiology, Institute of Environmental Medicine, Karolinska Institute, Visit: Nobelsväg 13, Box 210, 171 77 Stockholm, Sweden; 2 Laboratory of Population Health, Max Planck Institute for Demographic Research, Rostock, Germany; 3 Institute for Biodiversity and Ecosystem Dynamics, University of Amsterdam, Amsterdam, The Netherlands

**Keywords:** Life expectancy, Covid-19, Years of life lost, Care home, Multistate model, Excess deaths

## Abstract

The Covid-19 pandemic has not affected the population evenly. This must be acknowledged when it comes to understanding the Covid-19 death toll and answering the question of how many life years have been lost. We use level of geriatric care to account for variation in remaining life expectancy among individuals that died during 2020. Based on a linkage of administrative registers, we estimate remaining life expectancy stratified by age, sex, and care status using an incidence-based multistate model and analyze the number of years of life lost (YLL) during 2020 in Sweden. Our results show that remaining life expectancy between individuals with and without care differs substantially. More than half of all Covid-19 deaths had a remaining life expectancy lower than 4 years. Yet, in a 1-year perspective, Covid-19 did not seem to replace other causes of death. Not considering the differences in remaining life expectancy in the affected populations overestimated YLL by 40% for women and 30% for men, or around 2 years per death. While the unadjusted YLL from Covid-19 amounted to an average of 7.5 years for women and 8.6 years for men, the corresponding YLL adjusted for care status were 5.4 and 6.6, respectively. The total number of YLL to Covid-19 in 2020 is comparable to YLL from ischemic heart disease in 2019 and 2020. Our results urge the use of subgroup specific mortality when counting the burden of Covid-19. YLL are considerably reduced when the varying susceptibility for death is considered, but even if most lifespans were cut in the last years of life, the YLL are still substantial.

**Supplementary Information:**

The online version contains supplementary material available at 10.1007/s10654-022-00915-z.

## Introduction

It is clear that a large share of Covid-19 deaths have occurred among individuals with preexisting health problems [1–3]. This has led to speculations that deceased individuals would have died soon even in the absence of the pandemic. The controversy fueled debates about the effectiveness and costs of non-pharmaceutical interventions, such as lockdowns, considering also their potential unintended adverse consequences, including mental health problems [4–8]. An essential component of this discussion is the question to what extent Covid-19 and the further consequences of the pandemic have shortened lifespans, or in other words, how many life years have been lost because of the direct and indirect effects the Covid-19 pandemic.

Almost all countries experienced excess mortality during the pandemic [9, 10]. The increase in mortality has resulted in declining life expectancy across most countries where information is available [11, 12]. Both excess mortality as well as life expectancy have been argued to be among the most objective indicators for the consequences of the pandemic on population-level mortality [12–14]. Both indicators provide a net estimate of the mortality change, including additional deaths, such as those from Covid-19 itself but also other adverse health consequences of the pandemic, and averted deaths during the pandemic, such as potential lives saved due to the declining mobility [15].

In Sweden, life expectancy (LE) in 2020 has been estimated to be reduced by 0.6 years for females and 0.9 years for males compared to 2019 [12]. Considering that LE has increased continuously for decades, such a reduction is substantial.

An alternative perspective on the impact of the pandemic on mortality are years of life lost (YLL) [16–18]. YLL indicate the average remaining number of expected life years (remaining life expectancy) for an individual at the time of death. Life expectancies are usually drawn from life tables, and thus, reflect population-level estimates. Both the decline in life expectancy and the amount of YLL are the highest in Sweden compared to other Scandinavian countries. However, Sweden is no outlier if the comparison is extended towards other European countries, the US, or Brazil [12, 19, 20]. For Sweden, Kolk et al. [21] estimate 36,304 lost life years for women (7.99 years per death), and 48,174 lost life years for men (9.14 years per death) in 2020, while Pifarré I Arolas et al. [20] estimated 63,169 lost life years for Sweden with an average of 8.83 YLL for females and 10.28 for males using data until the end of November 2020. YLL estimates in both studies are based age- and sex-specific life expectancy combined with Covid-19 deaths.

Most studies that quantify the mortality impact of Covid-19 do not account for health status, and implicitly assume that individuals dying from Covid-19 had the same remaining life expectancy as the total population. However, in many countries including Sweden, the majority of deaths with Covid-19 took place among individuals receiving formal care, and thus, within a subpopulation, which has on average a poorer health status and lower remaining life expectancy compared to the total population [3, 22, 23]. Yet only a small number of studies have incorporated health information in the YLL estimation. For instance, Hanlon et al. [24] reported a decrease of YLL from 12 years to 9.4 years per death and 14 years to 11.6 years for women and men in Italy, while Ferenci [17] found a decrease from 10.5 to 9.2 years per death across Hungarian women and men. Both studies used the number and type of long-term health conditions as measure for health status. For Scotland, Burton et al. [25] found a reduction in total YLL from 7.05 to 4.88 years for women, and 7.39 years to 5.39 years for men, when distinguishing between care home residents and non-care-home residents.

In Sweden, municipalities are legally obliged to provide publicly funded geriatric care to older individuals in need. These services are allocated according to individuals’ needs. Most commonly individuals are offered home care, and only when a person’s needs can no longer be met in their own home do they move into a care home. This makes care status an important stratification criterion when analyzing life years lost in the context of Covid-19.

In this study, we investigate the death toll caused by the Covid-19 pandemic in Sweden along the dimensions of age, sex, and care status. We use life expectancy estimates derived from an incidence-based multistate model stratified by age, sex, and care status to analyze the questions of how remaining life expectancy differs between individuals with and without formal care, and how many life years have been lost due to Covid-19 when taking this aspect into account. To put the YLL into context, we additionally compare the YLL from Covid-19 to the YLL by other causes of death.

## Material and methods

### Data

Our study relies on different administrative population registers, which include all individuals registered in Sweden during the observation period (January 1, 2015–December 31, 2020) identified through the Total Population Register. Information on care status—distinguishing between receiving no formal care, receiving formal home care, and living in a care home—was derived from the Social Service Register (SSR). The SSR is an administrative register that collects monthly information on home care and care home residence [26]. Death dates and causes of death were identified from the Swedish Cause of Death Register.

Information on care status was only available for ages 70+. However, to provide a full picture of the Covid-19 death toll, we also incorporated deaths at ages below 70 into our analysis, for which we used aggregated annual death counts by age, sex and cause of death provided by Statistics Sweden [27]. For the estimation of excess mortality for ages below 70, we used weekly death counts by age and sex from the Short-term Mortality Fluctuations data series (STMF) and monthly population estimates from Statistics Sweden in addition to the register data that covered the ages 70+ [27, 28]. Life expectancy estimates for the total Swedish population are based on death counts and person years lived from the Human Mortality Database [29].

### Estimating life expectancy and YLL by care status

Care-specific life expectancy was calculated based on an incidence-based multistate model with rewards, described in more detail elsewhere [30]. This approach allowed us to model the transitions between the different care states, and to more accurately estimate the contribution of individuals that moved from one of the different care states to death. The multistate model was age-stage classified and included three transient states, which are receiving no care, receiving home care, and living in a care home. Based on the observation that improvements in the need for care were rare, our model did not include transitions back to a status with lower care demand. Death was the only absorbing state (see supplemental materials for more details).

Age- and birth cohort-specific probabilities of transitions between care states and of remaining in a care state were derived in intervals of three months for the range between ages 70 and 105 (see supplemental materials). The short interval allowed to capture several transitions of an individual within a short period. In the final multistate model, we used the median estimate from a set of 2000 smoothed transition probabilities that we obtained from fitting a Generalized Additive Model (GAM) with a P-Spline, an offset, and Poisson distribution to resample numbers of transitions [31]. To ensure that the probabilities at each age group sum up to one, we calculated the probability of staying in a state by subtracting the sum of all outgoing transitions from one. The resulting age-specific transition probabilities can be seen in the supplemental materials.

We used Markov chains with rewards to calculate the remaining life expectancy conditional on being in a specific state at a given age x. This methodology is based on the idea of assigning a specific reward to transitions, such as one if individuals survive an interval. From the respective transition and reward matrices, we calculated care-specific life expectancy following the methodology of Caswell and van Daalen [30]. To contrast the difference between YLL when considering care status and not, we also calculated life expectancy for the total Swedish population using standard life table methodology.

### Deaths for the calculation of YLL

We estimated YLL for deaths with Covid-19 as underlying cause of death (ICD-10 code: U071, U072, hereafter referred to as Covid-19 deaths) and all excess deaths, stratified by age, sex, and care status. In contrast to Covid-19 deaths that are based on death certificates, excess deaths indicate the net effect on mortality, including both additional and averted deaths during the pandemic. Deaths below age 70 were only available in 5-year age groups. We therefore used the Penalized Composite Link Model (PCLM) to ungroup the data and estimate age-specific counts [32, 33].

Excess deaths were defined as the difference between the observed deaths and deaths derived from baseline mortality. Baseline mortality—the hypothetical mortality level in absence of the pandemic—was estimated using a GAM with quasi-Poisson distribution to account for overdispersion. The model had three main components: (1) a component for the change in mortality over time, (2) a component for seasonality, and (3) an offset to incorporate changes in population size over time. The baseline mortality was estimated independently for each age interval (5-year age groups), sex, and care status (only at ages 70+), and the PCLM was used to obtain age-specific excess deaths. YLL were then calculated by multiplying the respective life expectancy and number of deaths at each age.

## Results

### Care-specific life expectancy

**Fig. 1:**
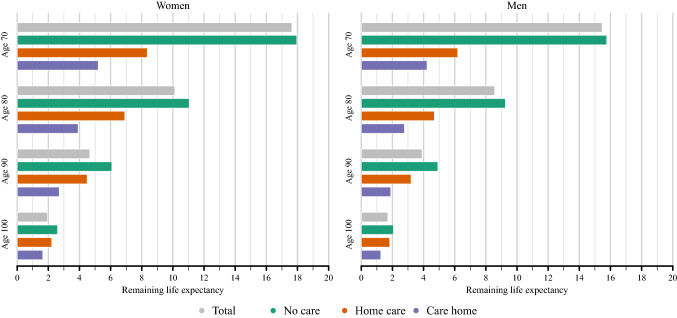
Life expectancy by care status and for the total population at different ages, women and men, Sweden, 2018–2019

Figure 1 exhibits care-specific and total life expectancy at ages 70, 80, 90, and 100 years separately for men and women. It illustrates the large differences in remaining life expectancy between individuals receiving care and those without care. Differences in life expectancy between the care groups are larger at younger ages and decline with age. At age 100, remaining life expectancy is between 1.5 and 2.5 years for women and 1–2 years for men depending on the care status, while differences in remaining life expectancy at age 70 are considerably larger with almost 18 and 16 years for women and men without care, but only 5 and 4 years for those living in a care home. At age 70, total life expectancy is very similar to the life expectancy of the no care population due to the very small share of individuals that receive care at this age (lower than 2% of the population). However, this share increases substantially with age. At age 90 and above, around 50% of men and 60% of women receive some form of care.

### Distribution of deaths by age, care status and remaining life expectancy

**Fig. 2:**
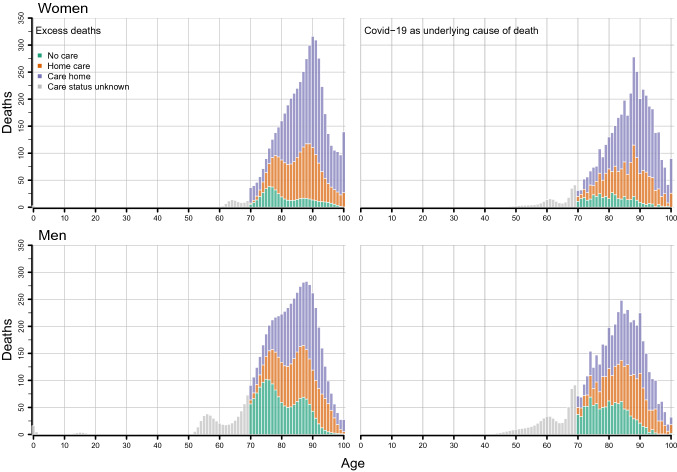
Distribution of deaths by age and care status separately for excess deaths and deaths with Covid-19 as underlying cause of death, men and women, 2020

Figure 2 shows the distribution of deaths over age. The panels on the left show excess death counts, while the right panels show deaths for which Covid-19 has been assigned the underlying cause of death separately for women and men. The figure exhibits several key findings. First, the distribution of excess deaths over age mirrors the distribution of deaths with Covid-19 as underlying cause of death, although death counts based on excess mortality are higher. Second, most deaths occurred among individuals who received care, either at home or in an institution. Third, in the group without care and below age 70, more deaths are observed among men than among women.

**Fig. 3:**
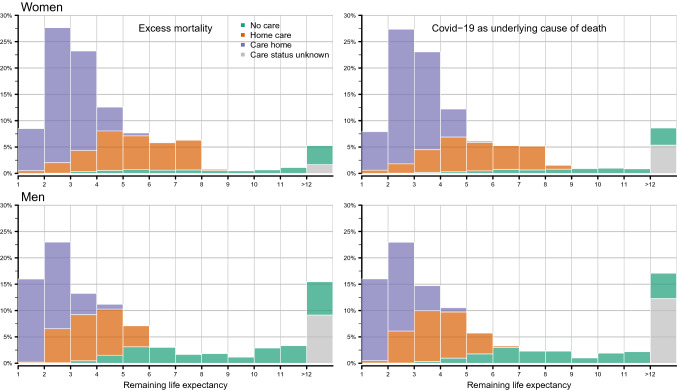
Distribution of deaths by remaining life expectancy and care status separately for excess deaths and deaths with Covid-19 as underlying cause of death, men and women, 2020 Notes: For deaths below age 70, where information on care status was unavailable, life expectancy for the total population was also used in the care-adjusted calculations for the total YLL

Figure 3 depicts the distribution of deaths by remaining life expectancy for excess deaths and for Covid-19 deaths. The gray bar depicts deaths with no information on care status (deaths below age 70). Deaths are grouped according to remaining life expectancy. The first group includes those with a remaining life expectancy between 1 and 2 years, while the last group includes those with a remaining life expectancy of 12 years or more. Note that the open-ended age group is 100+. For both sexes, remaining life expectancy is higher than 1 year in this group, and thus, there are no deaths with a life expectancy smaller than 1 year. The y-axis shows the percentages of all deaths that occurred in the respective group. The bars sum to 100%, and thus comprise all Covid-19/excess deaths.

The figure shows that for both men and women almost 60% of all deaths occurred among individuals with a remaining life expectancy less than four years. Among women, 7.5% of all Covid-19 deaths occurred among individuals with an expected remaining lifetime of 1–2 years. Among men the corresponding number is 16%. Less than 10% of all deaths for women and around 15% of all deaths for men had a remaining life expectancy of 12 years or more. Note that most deaths with unknown care status are deaths that occurred between ages 60 and 70 and most of them would likely belong to the no care group. With around 37.5% for men and 55% for women, a large share of deaths occurred in care homes.

Table 1 shows the number of deaths (excess and Covid-19 specific) and the respective YLL estimate using total life expectancy (unadjusted) and life expectancy adjusted for care state. The table also depicts the respective number of deaths and YLL by care status as well as the average number of YLL per death, which is shown in brackets.

**Table 1:** Death counts and years of life lost (YLL) in 2020, unadjusted and adjusted for care status for deaths with Covid-19 as underlying cause and excess deaths

	Total YLL (all ages)		Care-specific YLL (ages 70+)		
	Unadjusted	Adjusted	No care	Home care	Care home
*Women*					
C 19					
Deaths	4371	4371	402	1243	2491
YLL	32,581.6 (7.45)	23,575.7 (5.39)	4253.6 (10.58)	6553.3 (5.27)	7219.9 (2.90)
Excess					
Deaths	5152	5152	479	1623	2967
YLL	34,962.8 (6.79)	24,200.1 (4.69)	4964.3 (10.36)	8602.3 (5.30)	8552.2 (2.88)
*Men*					
C 19					
Deaths	5066	5066	1047	1474	1923
YLL	43,520.6 (8.59)	33,509.5 (6.61)	9824.4 (9.38)	5682.1 (3.85)	4419.8 (2.30)
Excess					
Deaths	6089	6089	1547	1721	2263
YLL	52,123.2 (8.56)	40,722.2 (6.69)	14,704.1 (9.50)	6635.1 (3.86)	5182.4 (2.29)

Average number of YLL per death shown in brackets. Notes: For deaths below age 70, where information on care status was unavailable, life expectancy for the total population was also used in the care-adjusted calculations for the total YLL

When adjusting for care status, the total number of YLL declines from 32582 to 23576 among women, and from 43,521 to 33,510 among men. Thus, not considering the differences in remaining life expectancy in the affected populations overestimated YLL by 40% for women and 30% for men. Consequently, the average YLL per death are around 2 years lower when care status is considered. Based on the adjusted average YLL per death and the distribution shown in Fig. 3, it becomes clear that around 70% of deaths had a remaining life expectancy that is lower than the average YLL per death.

For a woman who received no care and died from Covid-19, the estimated average YLL was 11 years, while the corresponding numbers were 5 and 3 years for women with home care or in a care home. For men, these numbers were 9 years, 4 years, and 2 years. The YLL were very similar for excess mortality.

**Fig. 4:**
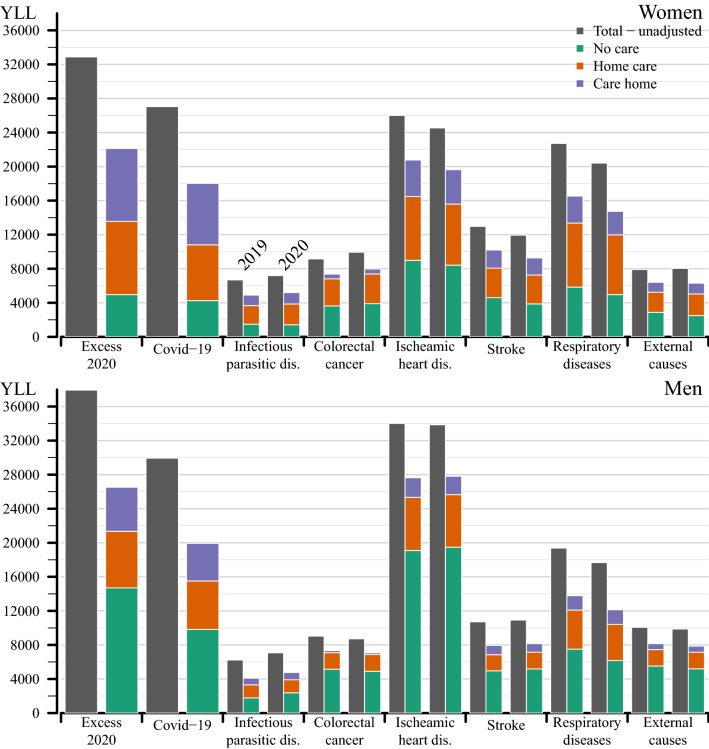
YLL for different causes of death in 2019 and 2020 and YLL from Covid-19 as underlying cause of death and excess mortality in 2020, ages 70+ , men and women Notes: Causes of death groups include infectious & parasitic diseases (ICD-10: **A**, **B**), colorectal cancer (C18–C21), ischemic heart disease (I20–I25), stroke (I60-I64), respiratory diseases (J), external causes of death (V, W, X, Y)

To put the YLL from Covid-19 in context, Fig. 4 shows YLL for excess mortality and Covid-19 deaths during 2020, as well as for some other causes of death during 2019 and 2020. For both men and women, the total number of YLL from excess mortality in 2020 was larger than all YLL from ischemic heart disease both in 2019 and in 2020. This is true also for Covid-19 mortality among women, while for men YLL to Covid-19 in 2020 were lower than YLL to ischemic heart disease. The lower bars in color show total YLL when care status is considered. The comparison between the grey and colored bars shows that considering care status matters more for Covid-19 mortality than for the other causes of death, although marked reductions are also visible for respiratory diseases and ischemic heart disease. It is also visible that none of the other causes of death in 2019 or 2020 come close to the share of YLL in care homes for excess mortality or Covid-19 mortality. Finally, by comparing the YLL for Covid-19 with other causes of death in the respective care groups, we see that YLL from Covid-19 in the home care group matches the single YLL from respiratory-, infectious-, and ischemic heart disease in the same care group.

## Discussion

We investigated the death toll of the Covid-19 pandemic in Sweden during 2020 along the dimensions of age, sex, and care status. We used care status-specific life expectancy in combination with confirmed Covid-19 deaths and excess deaths to estimate YLL in 2020 and compared it to other causes of death in 2019 and 2020. The comparison allowed us to put into context the amount of expected remaining life years that have been lost during the pandemic.

Our analysis revealed four key findings. First, remaining life expectancy varies greatly across care states. Second, the total amount of YLL from Covid-19 is reduced by 30% when care-specific life expectancy is considered, which translates to a reduction of around 2 years in the YLL per death. Third, more than 50% of all Covid-19 deaths had an average remaining lifetime of less than 4 years. Fourth, our results suggest that Covid-19 deaths did not replace other causes of death in 2020 but came on top of expected deaths. YLL from Covid-19 rank among the highest in comparison with other causes of death, also when considering care status.

Previous studies have documented a close link between existing comorbidities and an elevated risk for a severe progression and death from Covid-19 [1–3]. Our analysis of care status confirms these findings and additionally shows that a detailed understanding of the Covid-19 death toll requires information on health status, care need or similar variables that enables estimation of the variation in remaining life expectancy among those affected. Although the magnitude remains high, YLL are considerably reduced when care status is considered.

In Sweden as well as in other countries Covid-19 mortality has been particularly high among individuals in care homes [3, 22, 23], because they are frail, but also because they have been exposed to the virus to a greater extent than old people living at home. Protecting individuals within geriatric care has thus been a top priority in the public health response to the pandemic. The total number of YLL, especially among women receiving home care or living in care homes, however, show that protection efforts in Sweden have not been successful in the first year of the pandemic. Instead, the comparison to other causes of death shows that the Covid-19 pandemic was an unprecedented health threat to individuals receiving care.

The high number of YLL among men with no care is a worrisome finding. However, these numbers must be interpreted in the context of the gender differences in institutionalized care. Previous studies documented that living together with a potential caregiver considerably reduces the risk of admission to a care home [34, 35]. This likely also applies to the timing and health status at the onset of home care. Given that in the studied cohorts, married or partnered men are on average older than their wives, and women more often live alone, it is more likely that women act as care givers for their male spouses [36, 37]. This would result in an overestimation of the YLL among respective men without care since their remaining life expectancy at death would be somewhat lower than those measured for the no care group as a whole.

The observation that Covid-19 deaths closely resemble the distribution of excess deaths over age speaks towards that Covid-19 did not replace other causes of deaths but rather added deaths on top of the expected “normal” mortality. This is also confirmed when comparing the relative share of different causes on all deaths in 2019 and 2020 when excess deaths have been removed (see supplemental materials). The similar age distribution also shows that the pandemic particularly increased the mortality levels of frail individuals that already have a high death risk. The higher number of excess deaths compared to Covid-19 deaths may also suggest that cause-of-death-based death counts underestimate the true death toll of the pandemic, a problem that has been pointed out previously for many countries worldwide [38].

In most cases, Covid-19 deaths cut individual lifespans in the last years of life, as for instance, indicated by the finding that more than 50% of all deaths had an expected remaining lifetime of less than 4 years. This has implications for future mortality trends in Sweden. First, the concentration of deaths near the end of life suggests that potential mortality displacement may only have a short-term effect. We can thus assume that any adverse consequences following for instance from reduced cancer screening, or postponed care that may affect the total population will likely result in additional excess mortality. Second, increased mortality during 2020 will likely not result in a deviation of future mortality trends in the long run, even if the overall number of YLL caused by the pandemic remains high. Moreover, considering the variation in remaining life spans between affected groups, and the fact that many deaths had a remaining life expectancy well below the average number of YLL per death, this measure does not serve as a good summary indicator and should be used with caution in the case of estimating YLL during the Covid-19 pandemic for total populations.

Our paper has the strength of nationwide high quality administrative register data. This allowed us to use both cause of death statistics from medically signed death certificates, as well as excess mortality, an indicator for the adjusted net effect on mortality. We had access to monthly information about care status and could therefore model transitions in very short intervals, which is an advantage given the dynamics of transitions between care states and death. However, even with this approach, not all short-term transitions have been captured. Such transitions are mostly transitions into a state with higher care demand and into death soon after. Particularly remaining life expectancy for care home residence may thus be slightly overestimated. Moreover, we only had care status for the population aged 70 years and older. For ages below 70, we thus used total life expectancy as expected remaining lifetime, which may also lead to some overestimation of the remaining lifetime for some subgroups. There is likely also variation in death risk within different care status, which we did not consider in our analysis. With care-specific life expectancy, we thus capture variation between the different subgroups but not the variation within subgroups. This could result in an overestimation of YLL as individuals that died during 2020 may had a higher mortality risk compared to their subgroup peers. We also did not address the variation in the exposure to the virus within and across subgroups, which likely explains part of the differences in deaths and YLL between the different care groups. Individual health status and mortality risk would have captured even more variation in remaining life expectancy, yet also increased the complexity of the analysis. Overall, considering the large variation we observe in remaining life expectancy across the three care groups, we believe that our stratification of deaths and our estimates provide a more realistic picture of the YLL compared to previous estimates that stratify along age and sex only.

## Conclusion

The large differences in remaining life expectancy between individuals with and without formal care, and the fact that most Covid-19 deaths in 2020 in Sweden occurred in care homes or among individuals receiving home care illustrates the need for subgroup specific mortality rates when estimating the burden of Covid-19 on overall mortality. Nevertheless, despite the shorter life expectancies of many individuals that died from Covid-19, the data are in line with the view that Covid-19 did not replace other causes of death but were additional deaths that came on top of expected mortality, and thus, reduced lifespans in all groups. Even after taking care status into account, YLL to Covid-19 in 2020 remain comparable to YLL from ischemic heart disease.

## Supplementary Information

Below is the link to the electronic supplementary material.

Supplementary file1 (PDF 1047 KB)

## Data Availability

The study is partly based on national registers in Sweden and the datasets contain sensitive information. Access to the register data is available upon request from Karin Modig, given that the person interested to use it receives ethical vetting and approval from the data owners, the National Board of Health and Welfare in Sweden and Statistics Sweden. However, aggregated tables from the register data, freely available data used in the study, further resources, and all programming codes, which are needed to reproduce the presented results, are available from a public repository: https://osf.io/56u9q/(DOI10.17605/OSF.IO/56U9Q).
