# Supplementary material for: Years of life lost during the Covid-19 pandemic in Sweden considering variation in life expectancy by level of geriatric care
Source: Eur J Epidemiol. 2022 Sep 20;37(10):1025–34. doi: 10.1007/s10654-022-00915-z (PMC9488891; doi:10.1007/s10654-022-00915-z)
Supplement: Supplementary file 1 — Supplementary file1 (PDF 1047 KB) [file 10654_2022_915_MOESM1_ESM.pdf]

Supplemental materials: Years of life lost during the Covid-19 pandemic in Sweden considering variation in life expectancy by level of geriatric care

Marcus Ebeling[1,2] Enrique Acosta[2] Hal Caswell[3] Anna C. Meyer[1] Karin Modig[1]

March 14, 2022

1 Multistate model

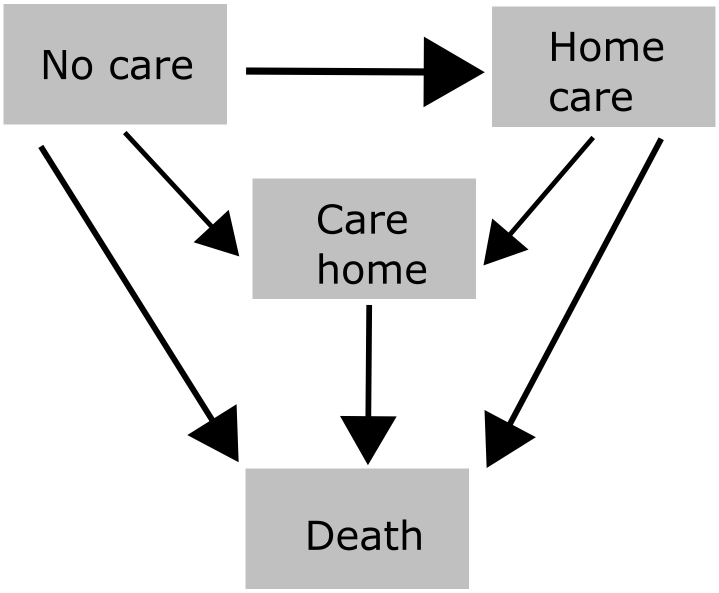

Figure 1: Specification of the mutlistate model used to estimate life expectancy by age and care status.

[1] Institute of Environmental Medicine, Karolinska Institutet, Stockholm, Sweden
[2] Max Planck Institute for Demographic Research, Rostock, Germany
[3] University of Amsterdam, Amsterdam, Netherlands

2 Transitions probabilities

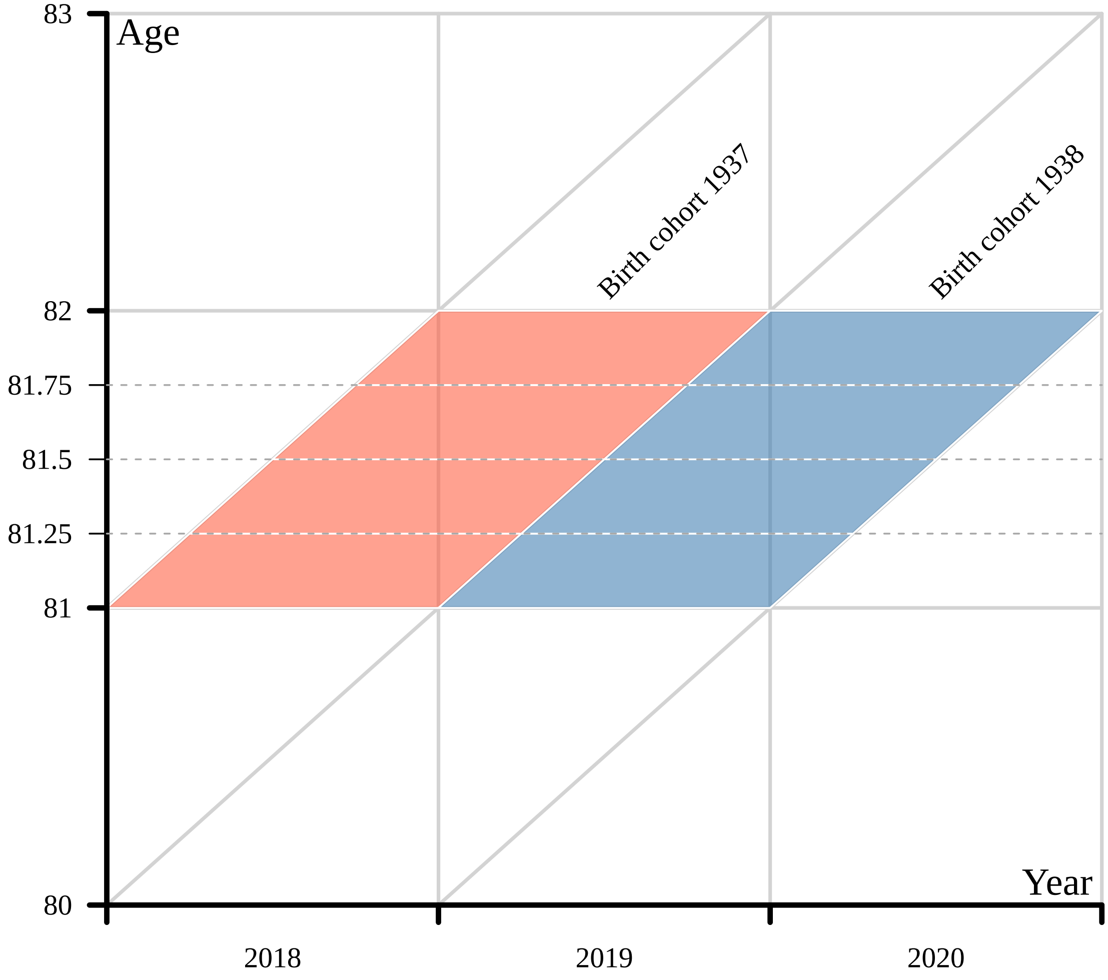

Figure 2: Illustration of the construction of age groups for the calculation of transition proabilities.

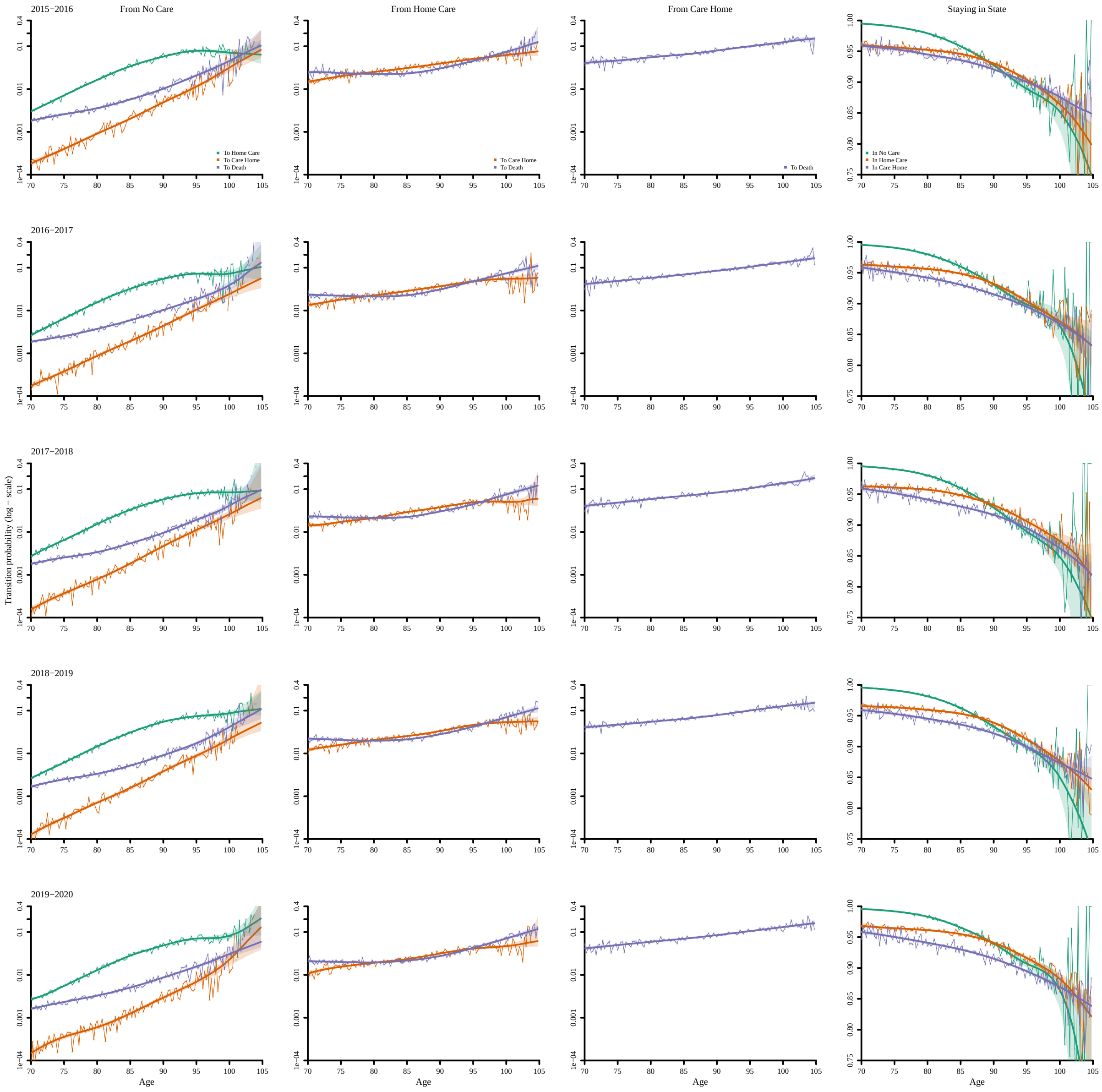

Figure 3: Observed and smoothed transition probabilities, Women, Sweden. Transition probabilities for the period 2018-2019 used as input for multistate model.

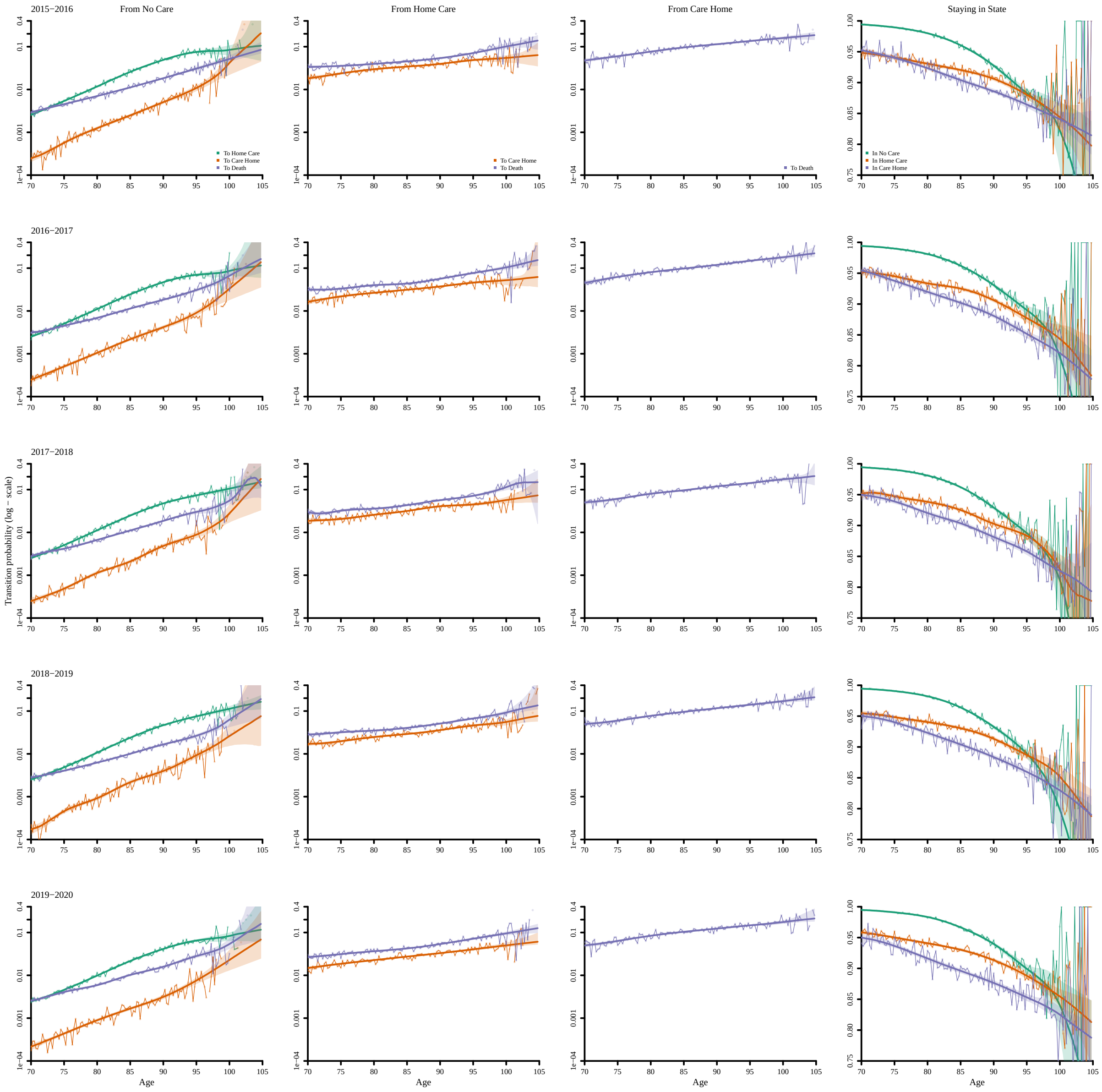

Figure 4: Observed and smoothed transition probabilities, Men, Sweden. Transition probabilities for the period 2018-2019 used as input for multistate model.

3 Sensitivity checks across alternative ways to calculate life expectancy from multistate model and between observed versus smoothed transition probabilities

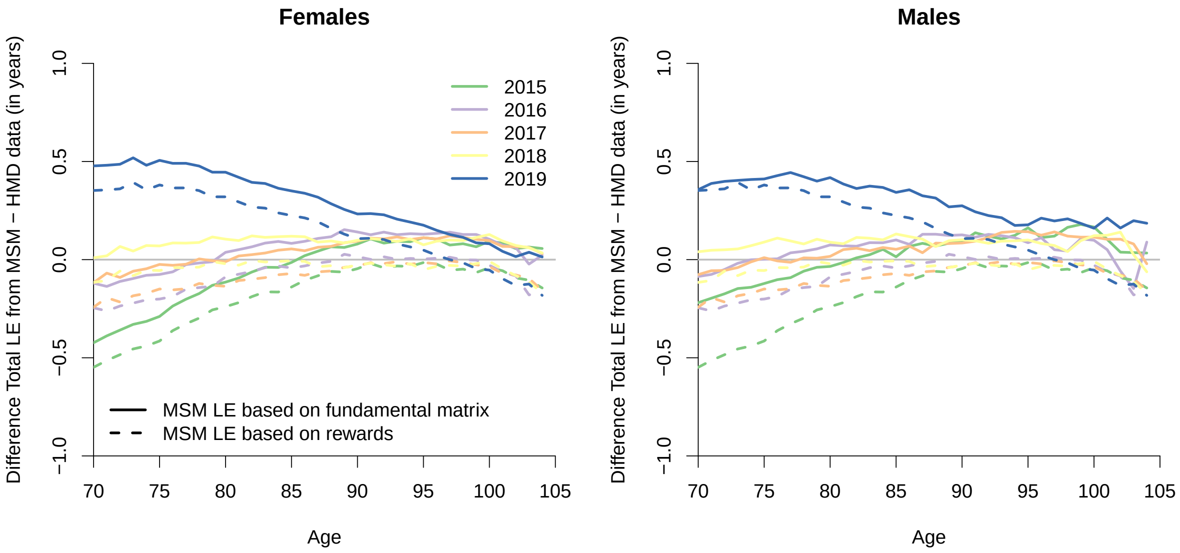

Figure 5: Differences between total life expectancy from multistate model and from life table calculation, Women and Men, Sweden.

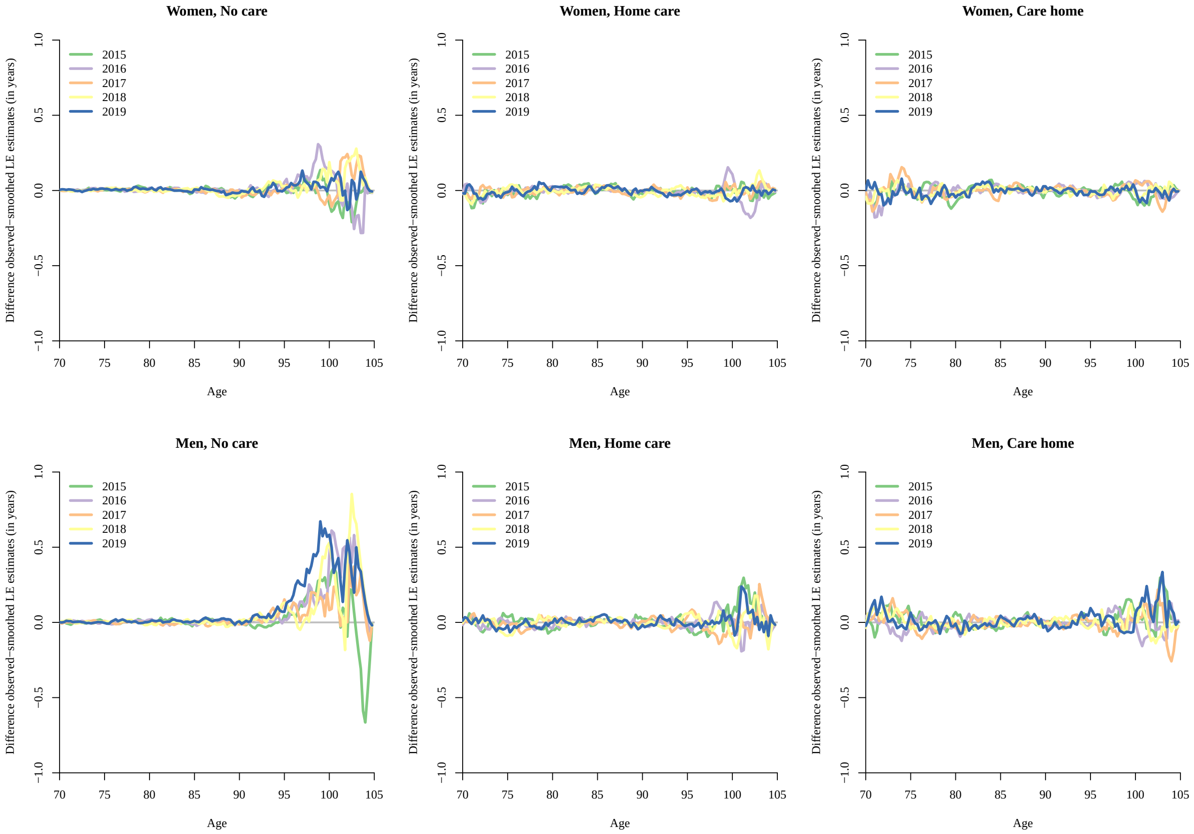

Figure 6: Differences between care-specific life expectancy based on observed and smoothed transition probabilities, Women and Men, Sweden.

4 Distribution of causes of death

Table 1: Proportion of deaths by different causes of death on the total number of deaths at ages 70 and above, women, 2019 and 2020, Sweden.

	Total			No care			Home care			Care home		
	2019	2020	2020*	2019	2020	2020*	2019	2020	2020*	2019	2020	2020*
Total deaths	38,678	42,698	37,629	6,824	7,183	6,704	14,123	15,432	13,809	17,731	20,083	17,116
Covid-19	–	9.69	–	–	5.60	–		8.05	–		12.40	–
Inf./parasitic dis.	2.53	2.49	2.83	1.99	2.03	2.18	3.01	3.05	3.41	2.35	2.23	2.61
Col. cancer	2.58	2.52	2.86	4.32	4.32	4.62	3.67	3.75	4.19	1.05	0.93	1.09
Isch. heart dis.	9.93	8.57	9.73	11.68	10.44	11.19	10.27	9.04	10.10	8.98	7.55	8.86
Stroke	4.72	3.99	4.53	6.40	5.08	5.44	4.66	4.21	4.70	4.11	3.43	4.03
Resp. diseases	7.33	5.90	6.70	7.05	5.74	6.15	9.18	7.75	8.66	5.98	4.54	5.33
External causes	2.96	2.77	3.14	3.77	3.15	3.37	3.29	3.21	3.58	2.39	2.29	2.69
All other	69.95	64.07	72.70	64.79	63.65	68.20	65.93	60.94	68.10	75.15	66.63	78.18

2020* - Excess deaths subtracted from total number of deaths

Table 2: Proportion of deaths by different causes of death on the total number of deaths at ages 70 and above, men, 2019 and 2020, Sweden.

	Total			No care			Home care			Care home		
	2019	2020	2020*	2019	2020	2020*	2019	2020	2020*	2019	2020	2020*
Total deaths	34,628	39,433	33,912	11,346	12,544	10,997	12,604	14,341	12,620	10,678	12,558	10,295
Covid-19	–	11.27	–	–	8.35	–	–	10.28	–	–	15.31	–
Inf./parasitic dis.	2.67	2.57	2.99	1.75	2.11	2.41	3.07	2.71	3.07	3.17	2.87	3.50
Col. cancer	2.99	2.52	2.93	4.28	3.60	4.10	3.51	3.15	3.58	1.01	0.72	0.88
Isch. heart dis.	13.12	11.39	13.25	16.90	15.32	17.48	12.42	10.97	12.46	9.92	7.95	9.69
Stroke	4.22	3.68	4.28	4.64	4.37	4.98	3.74	3.37	3.83	4.33	3.34	4.07
Resp. diseases	7.55	6.12	7.12	6.76	5.26	6.00	8.87	7.13	8.11	6.83	5.81	7.09
External causes	3.96	3.45	4.01	4.90	4.27	4.87	3.96	3.53	4.01	2.97	2.53	3.09
All other	65.49	59.01	68.64	60.76	56.72	64.70	64.42	58.87	66.89	71.77	61.47	74.98

2020* - Excess deaths subtracted from total number of deaths
